# Inhibition of *Campylobacter jejuni* Biofilm Formation by D-Amino Acids

**DOI:** 10.3390/antibiotics9110836

**Published:** 2020-11-23

**Authors:** Bassam A. Elgamoudi, Taha Taha, Victoria Korolik

**Affiliations:** Institute for Glycomics, Griffith University, Gold Coast QLD 4222, Australia; b.elgamoudi@griffith.edu.au (B.A.E.); Taha@griffith.edu.au (T.T.)

**Keywords:** D-amino acids, *Campylobacter jejuni*, biofilm, alanine racemase, confocal laser scanning microscopy

## Abstract

The ability of bacterial pathogens to form biofilms is an important virulence mechanism in relation to their pathogenesis and transmission. Biofilms play a crucial role in survival in unfavorable environmental conditions, acting as reservoirs of microbial contamination and antibiotic resistance. For intestinal pathogen *Campylobacter jejuni*, biofilms are considered to be a contributing factor in transmission through the food chain and currently, there are no known methods for intervention. Here, we present an unconventional approach to reducing biofilm formation by *C. jejuni* by the application of D-amino acids (DAs), and L-amino acids (LAs). We found that DAs and not LAs, except L-alanine, reduced biofilm formation by up to 70%. The treatment of *C. jejuni* cells with DAs changed the biofilm architecture and reduced the appearance of amyloid-like fibrils. In addition, a mixture of DAs enhanced antimicrobial efficacy of D-Cycloserine (DCS) up to 32% as compared with DCS treatment alone. Unexpectedly, D-alanine was able to reverse the inhibitory effect of other DAs as well as that of DCS. Furthermore, L-alanine and D-tryptophan decreased transcript levels of peptidoglycan biosynthesis enzymes alanine racemase (*alr*) and D-alanine-D-alanine ligase (*ddlA*) while D-serine was only able to decrease the transcript levels of *alr*. Our findings suggest that a combination of DAs could reduce biofilm formation, viability and persistence of *C. jejuni* through dysregulation of *alr* and *ddlA.*

## 1. Introduction

Human pathogen *Campylobacter jejuni* is a leading foodborne bacterial cause of diarrheal disease which, according to the World Health Organization (WHO), occurs annually in approximately 10% of the world’s population, including 200 million children [1,2]. Campylobacters are increasingly resistant to antibiotics; this is enhanced by their ability to form biofilms [3,4,5,6]. *C. jejuni*, in particular, is able to form mono- and mixed-culture biofilms in vitro and in vivo [7], which is recognized as a contributing factor of *C*. *jejuni* transmission through the food chain, where biofilms allow the cells to survive up to twice as long under atmospheric conditions and in water [8,9,10]. Campylobacters exhibit intrinsic resistance to many antimicrobial agents such as cephalosporins, trimethoprim, sulfamethoxazole, rifampicin and vancomycin, and are listed in WHO list of priority pathogens for new antibiotics development [3,4,11,12,13,14,15,16]. Biofilms are known to enhance antimicrobial resistance of many pathogens [3,4,5,17]; thus, unconventional approaches to controlling biofilms and improving the efficacy of currently used antibiotics are urgently needed. Recent investigations into potential antimicrobials include naturally occurring small molecules such as nitric oxide, fatty acids, and D-amino acids (DAs) [18,19,20,21]. DAs showed an ability to disperse some bacterial biofilms in vitro, such as those formed by *Bacillus subtilis*, *Staphylococcus aureus*, *Enterococcus faecalis* and *Pseudomonas aeruginosa* [22,23,24,25,26]. It is well documented that microorganisms preferentially utilize L-amino acids (LAs) over DAs [27,28], yet naturally occurring DAs have been found in different environments, such as soil, as well as in human and animals tissues [27]. In addition, many bacterial species secrete DAs in the stationary growth phase and when encased in biofilms. For example, Vibrio cholerae can produce D-methionine (D-met) and D-leucine (D-leu), while B. subtilis generates D-tyrosine (D-tyr) and D-phenylalanine (D-phe) which can accumulate at millimolar concentrations [29,30]. The ability of bacteria to produce DAs is proposed to be a mechanism for self-dispersal of aging biofilms, and DA production may also inhibit the growth of other bacteria during maturation of mixed biofilms. In a naturally occurring biofilm, DAs are found to be involved in the regulation of extracellular polymeric saccharide (EPS) production, for instance, D-tyr reduces the attachment of *B. subtilis,* *S. aureus* and *P. aeruginosa* to surfaces [23,31,32,33]. Also, DAs can induce disassembly of matrix-associated amyloid fibrils that link cells within the biofilm and contribute to the biofilm strength [34]. The effective concentration of DAs required to inhibit the biofilm formation varies depending on bacterial strain and DAs concentration ranging between 3 μM and 10 mM and D-Met, D-Trp and D-Ser were shown the most potent to inhibit the biofilm formation [24,33,35,36]. It is important to note that some DAs exhibit inhibitory or toxic effects on a number of bacterial species and can interfere with the activities of peptidases and proteases involved in cell wall synthesis, for example, D-met can be incorporated into the peptidoglycan (PG) of bacterial cell walls, causing morphological and structural damage [37].

DAs appear to be able to disrupt the biofilms via multiple mechanisms, offering an advantage to other biofilm dispersal agents which target a single process essential for biofilm formation, indicating that DAs could form the basis for a potential antibiofilm agent.

This study explores the effect of D and L amino acids, singly and in combination, on inhibition and dispersion of *C. jejuni* biofilms, the ability of these compounds to enhance the efficacy of antibiotics such D-cycloserine as well as potential mechanisms of inhibitory action. 

## 2. Results

### 2.1. Effect of LAs and DAs on Biofilm Formation by C. jejuni

**Figure 1:**
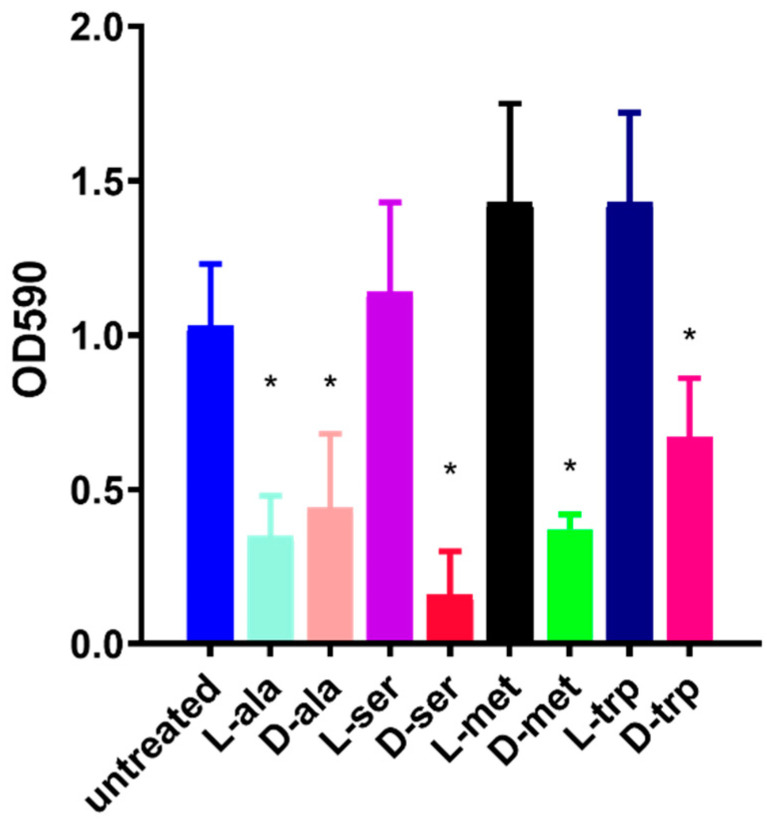
Effect of 100 mM D-amino acids (DAs) and L-amino acids (LAs) on *C. jejuni* 11168-O biofilm. Inhibition of biofilm formation in the presence of 100 mM of; L-alanine (L-ala), D-alanine (D-ala), L-serine (L-ser), D-serine (D-ser), L-methionine (L-met), D-methionine (D-met), L-tryptophan (L-trp), or D-tryptophan (D-trp). All data are mean ± Standard errors and were analyzed using an unpaired, two-tailed Student’s *t*-test, *p* < 0.05. The asterisk (*) indicates a statistically significant difference compared to untreated control.

In order to investigate the effect of LAs and DAs on biofilm formation, different concentrations of LAs and DAs (1–100 mM) were tested for their ability to disrupt or disperse the *Campylobacter* biofilm. Two assays were applied, one to measure the percentage of biofilm inhibition (%) (Inhibition Assay) and the other to determine the effect on the dispersion of a formed biofilm (Dispersion Assay). Treatment of *C. jejuni* culture with DAs showed significant inhibitory effect (*p*  < 0.001) on biofilm formation. Prescreening of individual LAs and DAs identified four (D-ala, D-met, D-ser, and D-trp) that had a potent ability to inhibit biofilm formation by *C. jejuni* (Figure 1). In contrast, the L-form of those amino acids, except L-ala, had no inhibitory effect, and L-met and L-trp, significantly increased biofilm formation.

**Figure 2:**
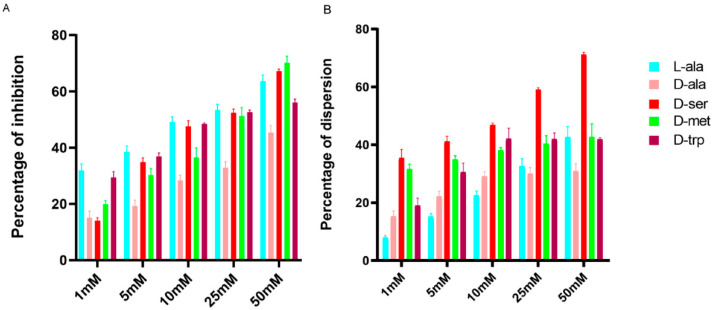
Inhibition and dispersion response of *C. jejuni* 11168-O biofilms in the presence of LAs and DAs at different concentrations. (**A**) Inhibition of biofilm formation by different concentrations of LAs and DAs, (**B**) Dispersion of the existing biofilm induced by different concentrations of LAs and DAs. The data is presented as Mean ± Standard errors of Percentage of inhibition (Normalized to untreated control).

The addition of DAs had a strong inhibitory effect on biofilm formation by *C. jejuni* in a dose-dependent manner (Figure 2) where 10 mM of D-trp reduced biofilm formation by 48% and 25 mM by 52%, while 10 mM and 25 mM D-ala reduced biofilm formation by 28% and 32%, respectively. Interestingly, 50 mM L-ala reduced biofilm by up to 63% as compared to 45% by D-ala at the same concentration (Figure 2). DAs had a disruptive effect on the existing biofilm where D-ser had the most significant effect (*p*  <  0.001) on formed biofilm disruption, up to 71%, at 50 mM compared to 1 mM or 10 mM (Figure 2), and the addition of 10 mM D-trp led to 42% disruption of formed biofilm.

Based on the determined concentrations of DAs required to elicit inhibitory or dispersal effect on biofilms, concentrations of various DAs and LAs between 2 to 25 mM were selected for subsequent assays.

### 2.2. Effect of DAs on Biofilm Formation by Different Campylobacter Strains

**Figure 3:**
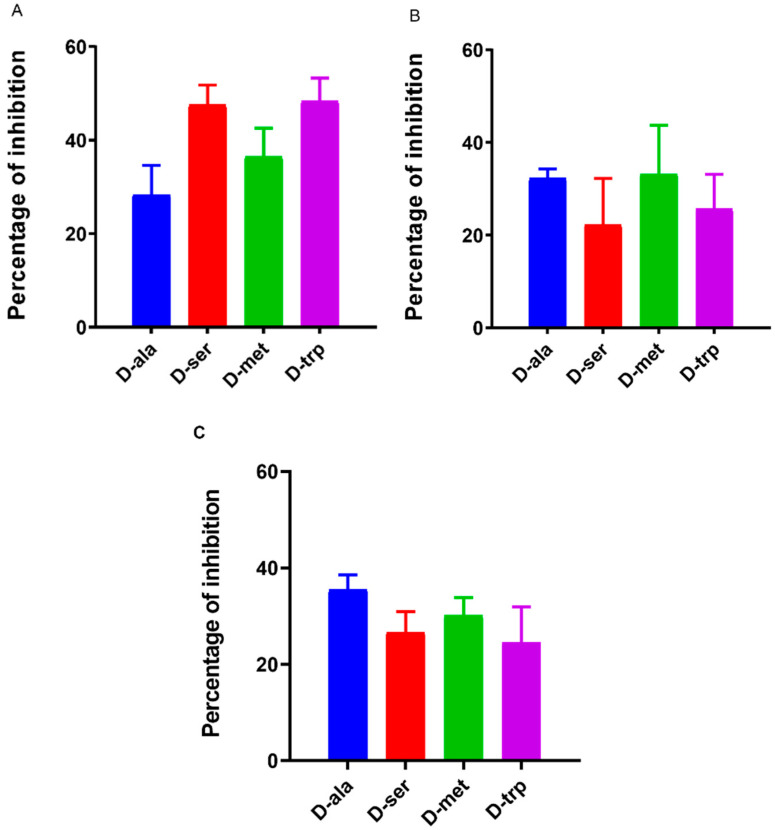
Quantitative analysis of biofilm inhibition of (**A**) *C. jejuni* 11168-O, (**B***) C. jejuni* 81-176, and (**C**) *C. coli* NCTC 11366 in the presence of 10 mM of DAs. The data is presented as Mean ± Standard errors of Percentage of inhibition (Normalized to untreated control).

In order to elucidate strain-specific responses, *C. jejuni* 11168-O, *C. jejuni* 81-176, and *C. coli* NCTC 11366, were used to confirm the inhibitory effect of D-ala, D-ser, D-met, and D-trp at 10 mM. The effect of DAs on biofilm formation was strain-dependent, whereas D-ser and D-trp had the greatest inhibitory effect on biofilm formation by 11168-O, D-ala and D-met were most effective against 81-176, and *C. coli* (Figure 3). 

### 2.3. Effect of the Equimolar Mixture of DAs and LAs on C. jejuni 11168-O Biofilm

**Figure 4:**
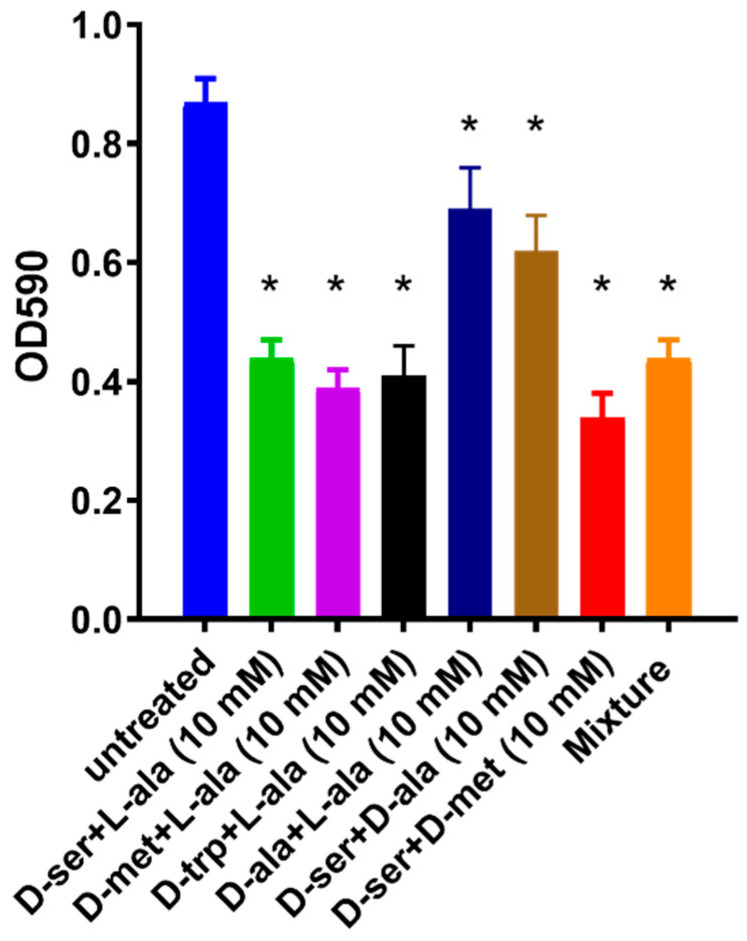
Effect of the equimolar mixture of DAs and LAs on *C. jejuni* 11168-O biofilm. All data are mean ± Standard errors and were analyzed using an unpaired, two-tailed Student’s *t*-test, *p* < 0.05. The asterisk (*) indicates a statistically significant difference compared to untreated control.

Considering that D-ser or D-met, applied at >1mM concentration, induced biofilm dispersal, while 5 mM L-ala, D-ser, D-met or D-trp had an inhibitory effect, equimolar mixture of DAs and LAs (1:1) was assessed. Equimolar mixture showed ≥40% inhibition of *C. jejuni* 11168-O biofilm formation (Figure 4). This suggested that using a combination of DAs and LAs, even at lowest concentrations, could be more potent than application of single DA or LA. The mixture of the four amino acids, L-ala, D-met, D-ser, D-trp was therefore assessed and a combination of these amino acids at 5:5:2:5 mM was more effective, with up to 49% inhibition of biofilm formation. Interestingly, the addition of D-ala decreased this inhibitory effect (Figure 4).

### 2.4. Microscopic Characterization of the Dispersion Effect of DAs on Biofilm

**Figure 5:**
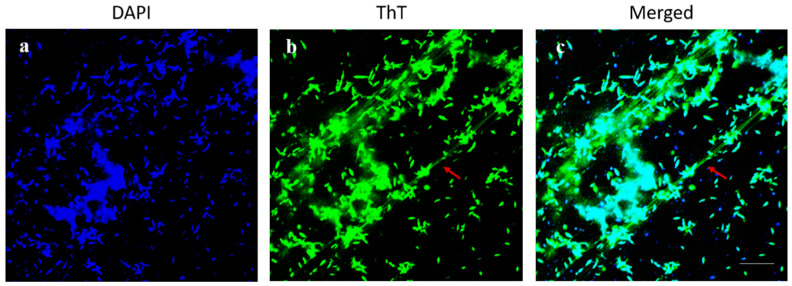
The mature biofilm of *C. jejuni* 11168-O and amyloid-like fibres. *C. jejuni* biofilm imaged using dual fluorescence labelling by confocal laser scanning microscopy (CLSM). (**a**–**c**) Bacterial cells within the biofilm (4′,6-diamidino-2-phenylindole (DAPI), blue) and red arrow indicates for amyloid-like *fibrils* (Thioflavin T (ThioT), green). (Scale bar = 10 µm).

Confocal microscopy showed that mature biofilm of *C. jejuni* 11168-O has structured appearance with amyloid-like fibrils which connect the cells within biofilms (Figure 5).

**Figure 6:**
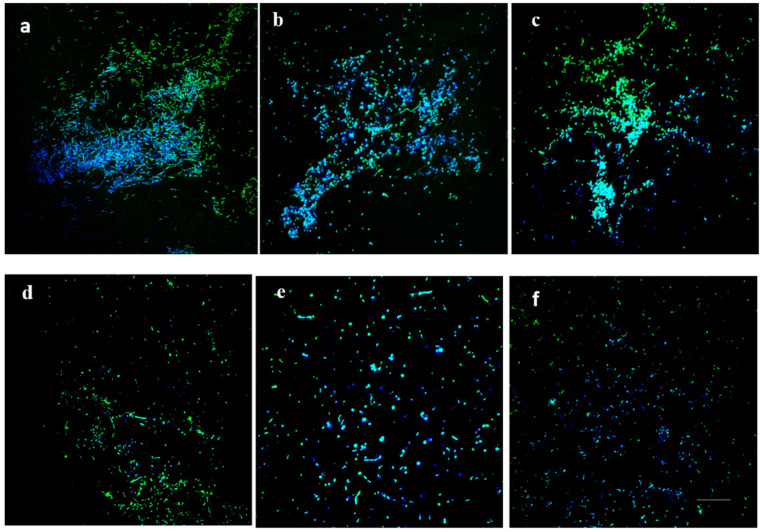
Confocal scanning laser microscopy images of *C. jejuni* 11168-O biofilm in presence of 25 mM of DAs. *C. jejuni* biofilm at 48 h, imaged using dual fluorescence labelling by confocal laser scanning microscopy (CLSM). (**a**) Untreated, (**b**) D-ala, (**c**) L-ala, (**d**) D-ser, (**e**) D-met, (**f**) D-trp. Cells were stained with 4′,6-diamidino-2-phenylindole (DAPI, blue) and amyloid fibrils by Thioflavin T (ThioT, green) (Scale bar = 20 µm).

Microscopic examination of formed biofilms, treated with individual DAs, showed a significant reduction in biofilm formation and disappearance of amyloid-like fibrils, compared to that of untreated controls (Figure 6). 

### 2.5. Expression Level of alr and ddlA in the Presence of LAs and DAs

In order to interrogate the mechanism of inhibitory action of DAs and L-ala, the expression of *C. jejuni* PG biosynthesis enzymes alanine racemase (*alr*) and D-Ala-D-Ala ligase (*ddlA*) in the presence and absence of DAs and LAs were examined. Therefore, 25 mM of DAs and LAs was chosen based on the inhibitory effects as shown in Figure 2. The relative expression of *ddl* and *alr* was downregulated by 1.25 to 4-fold below the cut-off level, respectively, following treatment of cells with 25 mM of L-ala (Table 1). In contrast, 25 mM of D-ala upregulated the expression of *ddl* by 10-fold and *alr* by 38-fold. Treatment of cells with 25 mM D-trp downregulated the expression level of *ddl* by 1.65-fold and *alr* by 3-fold whereas D-ser (25 mM) downregulated the expression of *alr* by 2.92-fold and upregulated *ddl* by 2.58-fold. No significant effect on the expression of *alr* and *ddl* was observed following treatment with D-met (Table 1). Interestingly, treatment of cells with D-Cycloserine (DCS) (10 ng/mL), as a positive control, had a greater effect, downregulating the expression of *ddlA* with a 7-fold change as compared to 2.85-fold change for *alr*. No loss of cell viability could be detected after 2 h exposure to DAs or DCS.

### 2.6. D-Ala Can Reverse the Inhibitory Effect of DAs and DCS

**Figure 7:**
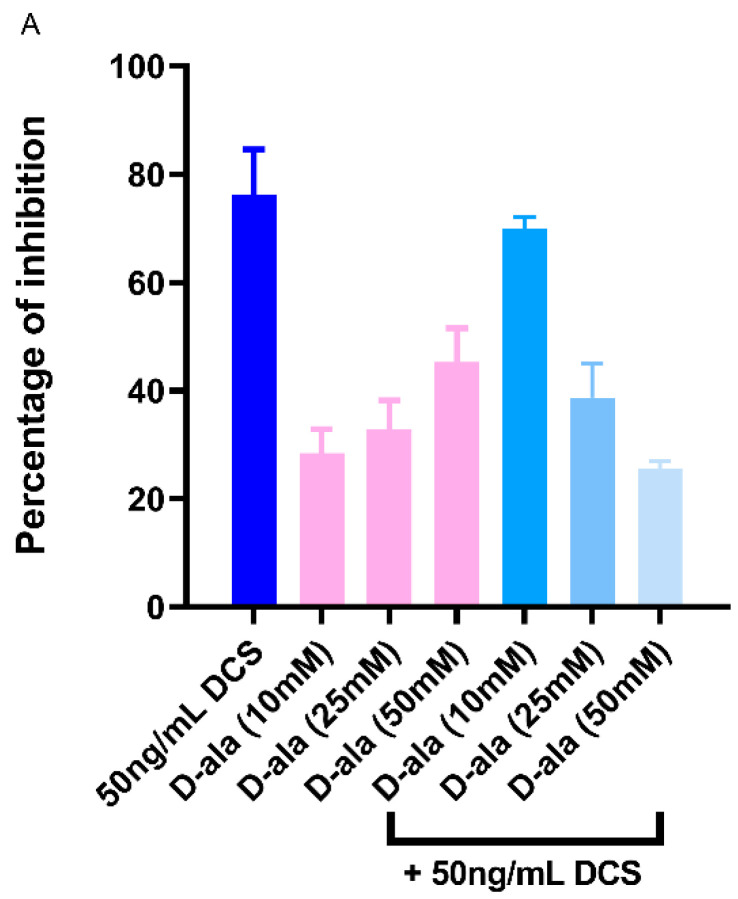

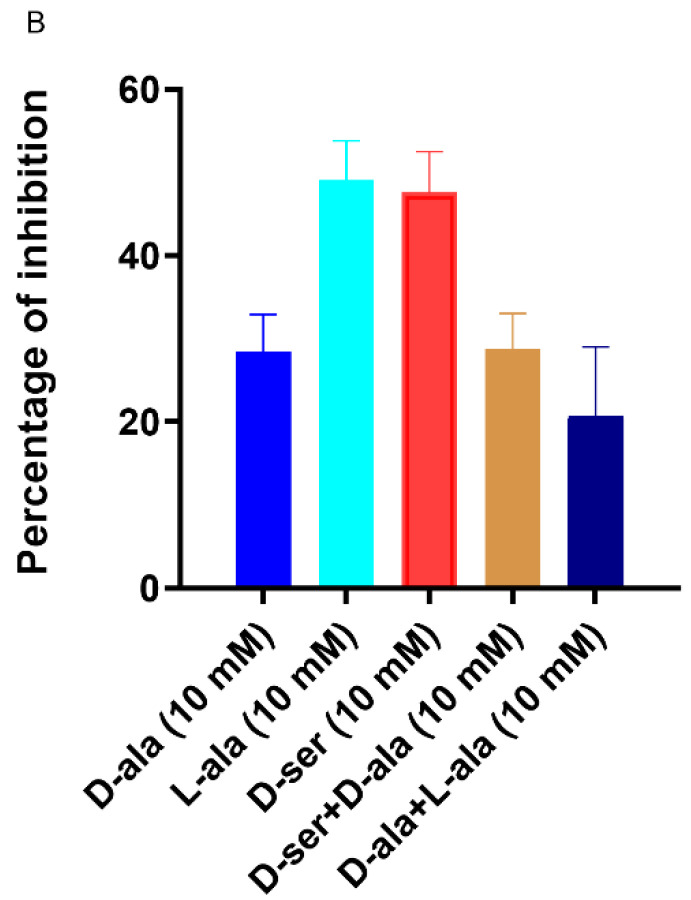
Reversal of *C. jejuni* 11168-O biofilm inhibition by (**A**) D-Cycloserine (DCS), (**B**) L-ala and D-ser in presence of D-alanine (D-ala). The data is presented as Mean ± Standard errors of Percentage of inhibition (Normalized to untreated control).

**Figure 8:**
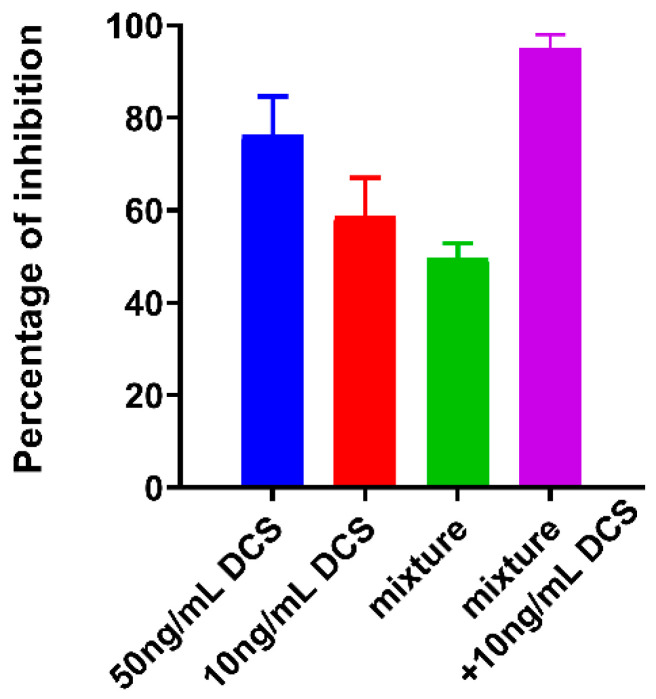
Effect of DCS on *C. jejuni* 11168-O biofilm when combined with a mixture of L-ala, D-ser, D-met, D-trp (5:5:2:5 mM). The data is presented as Mean ± Standard errors of Percentage of inhibition (Normalized to untreated control).

D-ala has been reported to reverse the antimicrobial efficacy of DCS in *Mycobacterium* spp. [38,39]. Considering that the minimum inhibitory concentration (MIC) range of DCS for *Campylobacter* spp reported being between 0.25–4 μg/mL [40], we tested the effect of sub-inhibitory concentration of 10–50 ng/mL DCS on *C. jejuni* cells and determined that DCS can reduce *C. jejuni* growth and biofilm formation by up to 60–76% (Figure 7 and Figure 8). Furthermore, this effect can be reversed by increasing the concentration of D-ala from 10 mM to 50 mM (Figure 7A). Combining D-ala with D-ser or with L-ala also decreased the inhibition of biofilm formation (Figure 7B). In contrast, a combination of DAs with DCS increased the efficacy of DCS at 10 ng/mL by 32% as compared with DCS treatment alone (Figure 8). 

## 3. Discussion

This study describes the activity of specific small, naturally occurring molecules, DAs, which are highly effective in preventing and disrupting *C. jejuni* biofilms, in concert with that previously shown for *B. subtilis, S. aureus* and *P. aeruginosa* [36,41]. While D-met and D-trp were able to inhibit the biofilm formation of *C. jejuni*, L-form of those amino acids significantly increased biofilm formation. It is possible that *C. jejuni* is able to catabolize L-form of those amino acids [42], which promotes bacterial growth, and consequently formation of the biofilm. This is consistent with the previous report of *B. subtilis* growth inhibition by D-form of Tyr, Leu, and Trp, and the L-form of those amino acids counteracting this effect [24]. The effect of DAs on inhibition and dispersal of *C. jejuni* biofilms showed a concentration-dependent response, with D-ser, D-met and D-trp being most effective in inhibition and dispersion of the biofilm. We observed that D-met, and D-trp, have a significant effect on triggering the disassembly of the biofilms at concentrations of ≥5 mM, similar to that observed for *S. aureus* and *P. aeruginosa* [43]. It is important to note that the inhibitory effect on the growth of *C. jejuni* by DAs, except D-met, could be reversed by D-ala, similar to that observed for *B. subtilis*, *M. tuberculosis* and *Escherichia coli* [38,39,44,45].

The microscopic analysis confirmed the effect of DAs on biofilm formation of *C. jejuni,* and particularly, the formation of amyloid-like fibrils within the biofilm matrix. Matrix-associated amyloid fibrils had been previously reported to form a part of *C. jejuni* biofilm [46], and similar DA-induced disassembly of matrix-associated amyloid fibers of *B. subtilis* biofilm, had been proposed as a biofilm-dispersal mechanism [34,41]. Together, these data allow us to speculate that the ability of DAs to promote the dispersal of formed *C. jejuni* biofilms, could involve the triggering the disassembly of matrix-associated amyloid fibrils.

While the mechanisms of antimicrobial and antibiofilm action of DAs, particularly, D-ser, D-met, and D-trp, are not fully understood, DAs effect on *C. jejuni* growth and biofilm formation may be similar to that for *Alcaligenes faecalis,* where D-met incorporates into PG, causing morphological and structural damage to the cell wall [30,37,47], and consequently suppresses bacterial growth. To explore that possibility, we interrogated the effect of DAs and LAs on the expression level of two genes in *C. jejuni*; alanine racemase (*alr*) (*Cj0905c*), and D-Ala-D-Ala ligase (*ddlA*) (*Cj0798c*) [48,49]. Both genes are encoding enzymes involved in an important step in D-Ala metabolism [44,50], which is essential for the synthesis of PG of the bacterial cell wall [45,51,52]. Two main reactions are involved in this process, first the conversion of L-Ala to D-Ala by alanine racemase (alr), and the formation of D-alanyl–D-alanine dipeptide (D-Ala-D-Ala) from D-ala by D-alanine–D-alanine ligase (ddl) [53]. RT-PCR data showed that DCS was able to reduce both *C. jejuni alr* and *ddlA* expression levels, similarly to L-ala, and D-trp. Interestingly, D-ser reduced *alr* expression levels, but not that of *ddlA*, suggesting that *ddlA* may not be the primary target for D-ser or DCS in *C. jejuni.* Furthermore, the ability of D-ala to reverse the inhibitory effect of DCS and D-ser suggests that the inhibitory effect of DCS and D-ser on *C. jejuni* can be mediated through inhibition of *alr* alone. In contrast, in *M. tuberculosis,* both *alr* and *ddl* were reported to be the primary targets of DCS [39], and Halouska et al. [54] suggested that *ddl* may be a primary target of DCS, rather than *alr*.

It is interesting to note that bacterial PG dipeptide D-Ala-D-Ala, which is generated by D-Ala-D-Ala ligase (*ddlA*), is the usual target for vancomycin, but in *C. jejuni,* PG contains D-Alanyl-D-Lactate (D-Ala-D-Lac) termini resulting in reduced efficacy of vancomycin by up to 1000-fold. Substitution by D-alanyl-D-serine (D-Ala-D-ser) termini reduces the efficacy of this antibiotic by up to 7-fold [4,55,56,57,58]. This further suggests that *alr* and not *ddlA,* is likely to be the primary target for D-ser and DCS in *C. jejuni*.

Our results suggest that DAs might have a promising application in enhancing of the activity antibiotics where the combination of DAs with DCS, synergistically increased the ability of DCS to inhibit *C. jejuni* biofilm formation and growth. The enhancement of DCS efficacy with DAs is likely to lower minimal dose requirement, which would consequently reduce the drug toxicity. DAs had also been reported to enhance the effectiveness for colistin and ciprofloxacin, when used against biofilms of *P. aeruginosa*, and rifampin used against biofilms of clinical isolates of *S. aureus* [43].

Here we have, therefore, demonstrated that D-alanine (D-ala),L-alanine (L-ala), D-serine (D-ser), D-methionine (D-met), and D-tryptophan (D-trp) can inhibit and disperse biofilms formed by *C. jejuni* and *C. coli* and that it may be possible to use these DAs to enhance the efficacy of antibiotics such D-cycloserine. Also, we presented evidence that DAs target alanine racemase (*alr*) in *C. jejuni,* which leads to the inhibition of growth and biofilm formation. This finding may be the key to understanding the mechanisms of DAs action and also could provide an alternative strategy to control *Campylobacter* spp transmission via the food chain.

## 4. Materials and Methods 

### 4.1. C. jejuni Strains and Growth Conditions

Bacterial strains used in this study were *C. jejuni* 11168-O (courtesy of Prof. D. G. Newell, Guildford, UK), *C. jejuni* 81-176 (courtesy of Prof. Christine Szymanski, University of Alberta, Edmonton, AB, Canada), and *C. coli* NCTC 11366 (Griffith University culture collection, Gold Coast, Australia). Cells were grown at 42 °C microaerobically (85% N_2_, 10% CO_2_ and 5% O_2_) on Mueller-Hinton agar (MHA) and in Mueller-Hinton broth (MHB), supplemented with Trimethoprim (5 µg mL^−1^) and Vancomycin (10 µg mL^−1^) (TV) (Sigma-Aldrich, Saint Louis, MO, USA). Microaerobic conditions were established by using Oxoid CampyGen (Thermo Scientific, Scoresby, Australia).

### 4.2. Chemical and Reagents Used in this Study

L-alanine (L-ala), D-alanine (D-ala), L-serine (L-ser), D-serine (D-ser), L-methionine (L-met), D-methionine (D-met), L-tryptophan (L-trp), D-tryptophan (D-trp) D- cycloserine were purchased from Sigma-Aldrich, Saint Louis, MO, USA. Individual stock solutions of 100 mM of DAs were prepared in Phosphate-buffered saline (PBS) (pH 7.2). 

### 4.3. Biofilm Formation and Dispersion Assays

Overnight cultures of *C. jejuni* strains were diluted to an OD_600_ of 0.05, and 2 mL of cell suspension were dispersed into 24-wells flat-bottom polystyrene tissue culture plates (Geiner Bio-One, Monroe, NC, USA). Different concentrations of DAs (1–100 mM) were added directly to the culture in the wells and incubated at 42 °C under microaerobic conditions for 48 h. For dispersion assay, *C. jejuni* cells were grown as described above, except no DAs were added. Then PBS containing the appropriate concentration of DAs (1–100 mM) was added to the wells and plates incubated for further 24 h. For crystal violet staining, plates were rinsed with water once (gently), dried at 55 °C for 30 min and stained using modified crystal violet staining method as described previously [59]. Data are representative of three independent experiments, and values are presented as Mean ± Standard errors. The percentage of biofilm inhibition and dispersion (%) was calculated as described in [60,61] by the following formula:

**%** = (control^OD590 nm^ − test^OD590 nm^/control^OD590 nm^) × 100. (1)

### 4.4. RNA Extraction, cDNA Synthesis and RT-qPCR of Alanine Racemase (alr), D-alanine-D-alanine Ligase (ddlA)

*C. jejuni* 11168-O cells were grown overnight microaerobically in MHB at 42 °C. Cells were collected by centrifuging at 4000 rpm for 15 min. The pellets were suspended in MHB and OD_600_ adjusted to 1 (~3 × 10^9^ cells/mL) and subsequently challenged with (1) 25 mM of L-ala, (2) 25 mM of D-ala, (3) 25 mM of D-ser, (4) 25 mM of D-met or, (5) 25 mM of D-trp for 2 h; (5) 10 ng/mL of DCS (below MIC which 250 ng/mL) was used as control. The bacterial survival was confirmed by viable cells counts after 2 h. Then, cells were collected by centrifugation at 4000 rpm for 15 min and pellets used for RNA extraction by RNeasy kit according to the manufacturer’s protocol (Bioline, Eveleigh, Australia). cDNA synthesis and RT-qPCR were performed as previously described [62]. The following primers sets were used: *alr* (Cj0905c) forward 3-AGCCAAAAATTTAGGAGTTT-5 and *alr* reverse 5-GAGGACGATGTGATAGTATT-3, *ddl* (Cj0798c) forward 3-TTATTTTTTGTGATGAAGAAAGAA-5 and *sdl* reverse 5-GAGTTCTTTTTCTTTTTTATAAGC-3. A *gryA* gene was used as a housekeeping control gene, using the primers, *gryA* forward 3-CCACTGGTGGTGAAGAAAATTTA-5 and *gryA* reverse 5-AGCATTTTACCTTGTGTGCTTAC-3. Relative *n*-fold changes in the transcription of the examined genes between the treated and non-treated samples were calculated using the relative quantification (RQ), also known as 2^−ΔΔCT^ method, where ΔΔ*C_T_* = Δ*C_T_* (treated sample) − Δ*C_T_* (untreated sample), Δ*C_T_* = *C_T_* (target gene) − *C_T_* (*gyrA*), and *C_T_* is the threshold cycle value for the amplified gene. The fold change due to treatment was calculated as −1/2^−ΔΔCT^ [63,64]. The data are presented as Mean ± S.D and were calculated from triplicate cultures and are representative of three independent experiments. 

### 4.5. Confocal Laser Scanning Microscopy

Overnight cultures of *C. jejuni* cells were diluted to an OD_600_ of 0.05, and 3 mL of each sample was placed into duplicate wells of a 6-well flat-bottom polystyrene tissue culture plate containing a glass coverslip to enable the formation of biofilm (Geiner Bio-One, Monroe, NC, USA). Thus, 25 mM of LAs and DAs were added directly to the wells, and then the plates were incubated at 42 °C microaerobically for 48 h. After the incubation, MH broth was removed, and the wells were gently washed with PBS solution twice to remove planktonic cells. The coverslips were carefully removed by using sterile needle and forceps to new 6-well plates and fixed using 5% formaldehyde solution for 1 h at room temperature. Then, the coverslips were gently washed with 2 mL of PBS and prepared for staining with fluorescent dyes.

### 4.6. Staining of C. jejuni Cells

The fluorescent DNA-binding stain DAPI (Sigma Aldrich, Saint Louis, MO, USA) was used to visualize cell distribution as described previously [65]. Thioflavin T (ThT) (Sigma Aldrich, Saint Louis, MO, USA) at 20 µM was then used to treat the coverslips for 30 min. ThT emits green fluorescence upon binding to cellulose or amyloids [66,67]. The coverslips then were mounted on glass slides using the mounting medium (Ibidi GmbH, Martinsried, Germany) and sealed with transparent nail varnish. Microscopy (Nikon A1R+) (Griffith University) was performed with two coverslips per sample from at least two separate experiments. All images were processed using ImageJ analysis software version 1.5i (National Institutes of Health, Bethesda, MD, USA).

### 4.7. Statistical Analysis

Statistical significance of data generated in this study was determined using two tailed Student’s *t*-test, GraphPad Prism (GraphPad Software version 8.0.0 for Windows, GraphPad Software, San Diego, CA, USA). *p* ≤ 0.05 was considered statistically significant.

## 5. Conclusions

To summarize, this study suggests that (i) DAs show the inhibitory effect at millimolar concentrations on biofilm formation by *C. jejuni*; (ii) DAs can trigger *C. jejuni* biofilm-disassembly; (iii) a combination of DAs can enhance the efficacy of DSC,(iv) DAs inhibit growth and biofilm formation of *C. jejuni* by repressing the expression of *alr.* The data described here contribute to the understanding of the mechanisms involved in biofilm dispersion and inform on identification of potential antimicrobial drug targets.

## Figures and Tables

**Table 1:** Analysis of the relative expression of *alr* and *ddlA* genes in the present of LAs and DAs by real-time PCR (RT-PCR). The relative expression of *alr* and *ddl* genes after incubation of *C. jejuni* 11168-O cells with 25 mM of LAs and DAs for 2 h.

	Fold Change						
	Upregulated			Downregulated			
Gene Name	D-ala	D-ser	D-met	L-ala	D-ser	D-trp	DCS
*alr*	38 ± 7	-	-	4.18 ± 0.3	2.92 ± 0.2	1.65 ± 0.3	2.85 ± 0.2
*ddlA*	10 ± 2	2.58 ± 0.6	-	1.25 ± 0.1	-	3.42 ± 0.4	7.15 ± 0.2
